# Fully Complementary Interactions of Human mRNA Genes with miRNAs from NCBI Database

**DOI:** 10.3390/cimb48100976

**Published:** 2026-09-23

**Authors:** Anatoliy Ivashchenko, Anna Pyrkova, Raigul Niyazova, Saltanat Orazova

**Affiliations:** 1 Department of Biotechnology, Farabi University, Almaty 050040, Kazakhstan; raygul.nyiyazova@kaznu.kz; 2 Center for Bioinformatics and Nanomedicine, Almaty 050040, Kazakhstan; anna.pyrkova@kaznu.kz; 3 Department of Computer Sciences, Farabi University, Almaty 050040, Kazakhstan

**Keywords:** miRNA, mRNA, human genes, nucleotides, diseases

## Abstract

miRNA has been studied for over 30 years, but the interaction of miRNA with mRNA has not been sufficiently studied to date. Problems in this area of research include the lack of adequate methods for predicting the characteristics of miRNA-mRNA interactions, and wet lab experiments are many times more labor-intensive and time-consuming. The aim of this study was to determine the characteristics of fully complementary interactions of miRNAs from the 2568 miRNAs of the NCBI database with mRNAs of 17,508 protein-coding genes using the MirTarget program. It was found that mRNAs from 384 genes have binding sites (BSs) with 89 miRNAs. miR-619-5p has 220 BSs in mRNA from 201 genes. mRNA from 17 genes has two BSs for miR-619-5p and the mRNA of the *CACNG8* gene has three BSs. Three mRNAs contained miR-619-5p BSs in the 5′UTR, only one gene had a CDS, and the remaining BSs were in the 3′UTR. miR-5096 binds to the mRNAs of 45 genes, 12 of which also bound miR-619-5p. *IL18* mRNA bound two miR-5096s. MiR-1273f targeted 12 genes, miR-1273g-3p targeted nine genes, and miR-1273h-5p targeted four genes. MiR-1548ap-3p, miR-1285ap-5p, and miR-4478 each had four target genes. Several pairs of miRNA-5p and miRNA-3p were bound to mRNAs of the *RTL*, *CCDC42B*, *FOXF2*, *GLYCTK*, *KAA2026*, and *LPPR3* genes. The highest free energy of interaction was found for miR-4665-3p (−159 kJ/mol), miR-1-356-5p (−146 kJ/mol), and miR-1-155-3p (−138 kJ/mol). miRNAs from the NCBI database can significantly suppress the translation of many mRNAs. The obtained data show reliable links between miRNAs and target genes that can be used to diagnose diseases caused by disturbances in these interactions.

## 1. Introduction

miRNAs (mRNA inhibiting RNA) that bind to mRNA of genes and suppress protein synthesis were discovered more than 30 years ago [1]. Nanosized miRNAs, the length of which varies from 18 nucleotides (5.8 nanometers) to 25 nucleotides (8.5 nanometers), are logically called miRNA but not microRNA. miRNAs participate in the regulation of the synthesis of many proteins and are regulators of the expression of most cellular genes [2,3,4,5,6,7,8]. Identifying miRNA target genes in wet assays is costly and time-consuming. Because miRNA-mRNA interactions are seemingly simple processes, numerous programs have been proposed to predict these interactions. Programmers relied on the unfounded assumption that miRNAs could interact using only 6–9 nucleotides, the so-called “seed” region. This misconception persisted for over thirty years until it was convincingly demonstrated that miRNA-mRNA interactions must involve the entire miRNA nucleotide sequence. Another feature of miRNA involvement in diseases is that several miRNAs can interact with one mRNA and one miRNA can interact with several mRNAs, which is clearly demonstrated in the publications [2,9,10,11,12,13]. Therefore, many publications have essentially only established correlations between changes in miRNA concentration and the amount of synthesized protein or disease development. Since many diseases are associated with disrupted expression of protein-coding genes, the involvement of miRNA in these processes requires studying the direct interaction of miRNA with mRNA, preferably with quantitative characteristics of this interaction. The use of inadequate methods for establishing specific miRNA-mRNA relationships involved in these processes has not led to the development of reliable diagnostic and therapeutic methods using miRNA. The goal of this study is to demonstrate that many miRNAs can interact fully complementarily with mRNA and, therefore, can play an important role in regulating human gene expression.

## 2. Materials and Methods

The nucleotide (nt) sequences of 17,508 human genes were downloaded from the National Center for Biotechnology Information (NCBI) (https://www.ncbi.nlm.nih.gov (accessed on 1 January 2022)). The mRNA isoforms with the maximum nucleotide length were used. The nucleotide sequences of the 2567 miRNAs were taken from the miRNA database of NCBI (http://mirbase.org (accessed on 1 January 2022)). The miRNA binding sites (BSs) in mRNA were predicted using the MirTarget program (version 2.0) [14]. This program predicts the following features of miRNA binding to mRNA: (a) the initiation of miRNA binding to the mRNA from the first nucleotide of the mRNA; (b) the localization of the miRNA BSs in the 5′-untranslated region (5′UTR), coding domain sequence (CDS), and 3′-untranslated region (3′UTR) of the mRNAs; (c) the schemes of nucleotide interactions between miRNA with mRNA, which clearly demonstrate the interactions of all nucleotides between miRNA with mRNA; (d) the free energy Gibbs (ΔG, kJ/mol) of the interaction between miRNA and the mRNA; and the ratio ΔG/ΔGm (%) is determined for each site. ΔGm equals the free energy of miRNA binding with its fully complementary canonical nucleotide sequence. Only miRNAs whose nucleotides interacted with mRNA using canonical (G-C and A-U) nucleotides with a given ΔG value were selected from the calculated data. The MirTarget program finds hydrogen bonds between miRNAs and mRNA according to the physicochemical characteristics of nucleotide interactions [15,16,17,18]. The MirTarget program differs from other programs in terms of finding miRNA BSs on mRNA in the following ways: it takes into account the interaction of miRNA with mRNA over the entire miRNA nucleotide sequence, and it calculates the free energy of the interaction of the miRNA with mRNA. Note that the G, A, C, and U nucleotides, which comprise the RNA structure of microorganisms, plants, and animals, interact identically under equal conditions. The difference between the MirTarget program and other programs is given in Figure 1.

Therefore, the physicochemical properties of canonical nucleotide pairs given above do not require additional proof of the previously established physicochemical characteristics of their interaction. The reliability of translation suppression by miRNAs that are fully complementary to mRNAs was proven by Fire A.Z. and Mello C.C., who were awarded the Nobel Prize in 2006 for this research. Biologists Ambros V. and Ruvkun G. were awarded the Nobel Prize in 2024 for similar research.

## 3. Results

In this study, we identified miRNAs that bind fully complementarily to mRNAs from 17,508 human genes in the NCBI database. The largest number of target genes was identified for miR-619-5p, which had 220 binding sites in 201 genes (Table 1). The mRNA of the *CACNG8* gene had three miR-619-5p binding sites located in the 3′UTR. The *CACNG8* gene is weakly expressed in the stomach, heart, and kidney, and therefore is under strong control of miR-619-5p. The mRNA of seventeen genes had two miR-619-5p BSs each: *C17orf75*, *C8orf44*, *CHST6*, *CIAO1*, *CPM*, *CYP20A1*, *DCAF10*, *FKBP14*, *GK5*, *KREMEN1*, *LMOD3*, *RAB3IP*, *SYNJ2BP*, *TMEM213*, *TRPM7*, *VHL*, *XIAP*, and *ZNF667*. miR-619-5p has an intronic origin from the pre-miRNA of the *SSH1* gene, which is expressed in many tissues, and therefore, miR-619-5p may also be present in many tissues and influence the expression of its target genes in them. Many of these target genes are expressed to a higher degree than the *SSH1* gene, and therefore miR-619-5p cannot exert significant control over the synthesis of the corresponding proteins. However, due to the general dependence on miR-619-5p, a balance is established between the protein synthesis of the target genes of this miRNA. Many miR-619-5p target genes have been identified for a long time [2], but this miRNA has not attracted much interest from researchers. Clearly, controlling the influence of miR-619-5p on such a large number of genes is very difficult, but it is necessary, since this unique phenomenon is highly likely not random and can explain many physiological properties of humans, given the wide range of functions of the target genes.

miR-5096 has 45 target genes, of which only the mRNA of the *IL18* gene had two BSs (Table 2). All miR-5096 BSs were localized in the 3′UTR. The expression of the 45 miR-5096 target genes may likely be linked via this miRNA, since increased expression of one gene will lead to increased binding of miR-5096 to its mRNA, and the expression of other target genes will increase. Twelve miR-5096 target genes have BSs for miR-619-5p, which increases the control of these genes’ expression by miRNA. Of these, in the mRNAs of the *C15orf40*, *LMOD3*, *PNPLA1*, and *ZNF197* genes, the beginning of the miR-5096 BS is located 74 nt before the beginning of the miR-619-5p BS. This arrangement of the BSs for miR-5096 and miR-619-5p is not random and reflects the relationship between these miRNAs and their target genes.

Expression of several genes depends on pairs of miRNAs (miRNA-5p and miRNA-3p) that originate from the same pre-miRNA hairpin. We have previously identified quantitative characteristics of their interactions with target genes [17,18]. This surprising property is possessed by mRNA of the *RTL1* gene, containing five pairs of binding sites for miR-127-3p and miR-127-5p, miR-136-3p and miR-136-5p, miR-431-3p and miR-431-5p, miR-432-3p and miR-432-5p, miR-433-3p and miR-433-5p. Schemes of interaction of these miRNAs with quantitative characteristics are shown in Figure 2. All binding sites for these miRNAs were located within the CDS of the *RTL1* gene mRNA at intervals of 34 nt in the miR-127-3p and miR-127-5p pair, 33 nt in the miR-136-3p and miR-136-5p pair, 44 nt in the miR-431-3p and miR-431-5p pair, 46 nt in the miR-432-3p and miR-432-5p pair, and 52 nt in the miR-433-3p and miR-433-5p pair. Importantly, the miRNA nucleotide sequences were identical in animals that diverged millions of years ago.

Each of the mRNAs from the genes *CCDC42B*, *FOXF2*, *GLYCTK*, *KAA2026*, and *LPPR3* bound a pair of miRNA-5p and miRNA-3p (Figure 3). The data show that the binding sites for these miRNA pairs were localized in the CDS, 3′UTR, and 5′UTR. For these genes, experiments showed that the presented results of computer analysis were fully confirmed [18]. Our results add to them the free energy of interaction between miRNA and mRNA. The beginnings of miRNA binding sites in the pairs miR-7106-5p and miR-7106-3p, miR-6720-5p and miR-6720-3p, miR-135a-5p and miR-135a-3p, miR-4665-5p and miR-4665-3p, and miR-3187-5p and miR-3187-3p were located at 40 nt, 35 nt, 38 nt, 39 nt, and 34 nt, respectively.

miRNA-1-155-3p has ten target genes (Table 3). The mRNAs of six genes contain binding sites in the 5′UTR, while four have sites in the CDS. This distribution is likely due to the higher GC content of miRNA-1-155-3p and the target mRNAs. This high GC content is naturally reflected in a strong interaction free energy of −138 kJ/mol. Similarly, miRNA-1-356-5p features three binding sites within the 5′UTRs of two target genes, including *BMP8B*, which harbors two binding sites spaced just one nucleotide apart. This mRNA–miRNA pair exhibits an even lower (more favorable) interaction free energy of −146 kJ/mol. Notably, miR-762 also targets *BCL7C* and *TBC1D10B* with an interaction free energy of −136 kJ/mol, indicating a strong potential for translational repression.

miR-155 is a well-established regulator of immunity and cancer, and there is increasing evidence that its 3p strand plays a role in these arenas.

The miR-548 family has binding sites in the mRNA of five genes (Table 4). The mRNAs of the *ALG10* and *SMU1* genes each had one miR-548 binding site. The mRNAs of the *ANKRD5* and *TCEANC2* genes each had three miR-548 binding sites, and the mRNA of the *KATNAL1* gene had six miR-548 binding sites. All binding sites for these miRNAs are located in the 3′UTR. The free energy of interaction of these miRNAs with mRNA varied from −89 kJ/mol to −123 kJ/mol, which depended on the GC content and the length of the miRNA. If the miRNA binding sites in mRNA were located with overlapping nucleotides, we called such mRNA regions binding site clusters. In such clusters, competition between miRNAs for binding to mRNA occurs. Three miRNAs from the miR-548 family bound in a cluster at 3534 nt, and three miRNAs at 3495 nt. Notably, the effect of miR-762 on the *BCL7C* and *TBCID10B* genes, with a free energy of interaction of −136 kJ/mol. This interaction ensures strong suppression of the synthesis of the corresponding proteins.

Members of the miR-1273 family play a significant role in the regulation of gene expression (Table 5). miR-1273f affects 12 target genes, each of whose mRNAs contains one BS of this miRNA. In all but one of these mRNAs, the BSs are located in the 3′UTR. miR-1273f consists of 19 nucleotides, and the free energy of interaction with the mRNA of the target genes is −104 kJ/mole (Table 5). BSs of miR-1273g-3p are located in the 3′UTR of the mRNA of nine genes. mRNA of four genes is the target of miR-1273h-5p (Table 5). The free energy interaction of miR-1273g-3p and miR-1273h-5p with the mRNA of the target genes is −117 kJ/mole at a length of 21 nucleotides. In total, the miR-1273 family targets 25 different genes, making it difficult to elucidate the biological role of these miRNAs.

miR-1184 bound to mRNA of three different genes, and miR-1285-5p bound to mRNA of four genes (Table 6). The family of five miR-1260s bound to mRNA of five genes in the CDS and 3′UTR. miRNA-3178 and other miRNAs had binding sites in individual genes. Table 6 highlights miRNA-4478, which has four target genes, and miRNA-4508 and miRNA-5095, which each have three target genes. The total number of miRNAs identified in this study between 18 and 24 nucleotides in length was 310, of which 21- and 22-nucleotide miRNAs accounted for 82%, ensuring highly selective miRNA and mRNA interactions.

For identical miRNA lengths, the free energy of interaction (ΔG) between miRNA and mRNA depends on the GC content, and for identical miRNA GC content, the free energy of interaction depends on the miRNA length.

## 4. Discussion

A previous publication demonstrated that 201 target genes with miR-619-5p binding sites are involved in numerous metabolic reactions, including those associated with various diseases [2]. Therefore, the proposed biological role of miR-619-5p and other miRNAs with multiple target genes is to maintain a balance between the expression of the target genes in this association. Increased mRNA synthesis for one gene will result in preferential miRNA binding, and other genes will show a slight increase in expression while the amount of this miRNA remains constant. We found that not only can one miRNA bind to an mRNA, but other miRNAs can bind to the mRNA in a fully complementary manner. It has been established that multiple miRNAs can bind to a single region (cluster of binding sites) of mRNA, competing with each other, indicating robust control of gene expression. It has been shown that mRNAs of different genes contain identical binding clusters for identical miRNAs. The use of a specific miRNA for diagnosis or therapy necessarily requires identifying the effect of this miRNA on other genes to avoid side effects of the miRNA used [19,20,21]. A miRNA can suppress oncogenesis if it acts on an oncogene and can stimulate oncogenesis if the miRNA acts on a tumor suppressor. The provided list of properties of miRNA-mRNA interactions demonstrates the need to consider the above-mentioned features of miRNA-mRNA interactions. Since many diseases are associated with impaired expression of protein-coding genes, the involvement of miRNA in these processes requires confirmation of the interaction of miRNA with mRNA, preferably with specific characteristics of this interaction. The use of inadequate methods for establishing specific miRNA and mRNA relationships involved in these processes has not led to the development of reliable diagnostic and therapeutic methods using miRNA. The present studies have shown that many miRNAs can interact fully complementarily with mRNA and, therefore, can play an important role in the regulation of human genome expression.

## Figures and Tables

**Figure 1:**
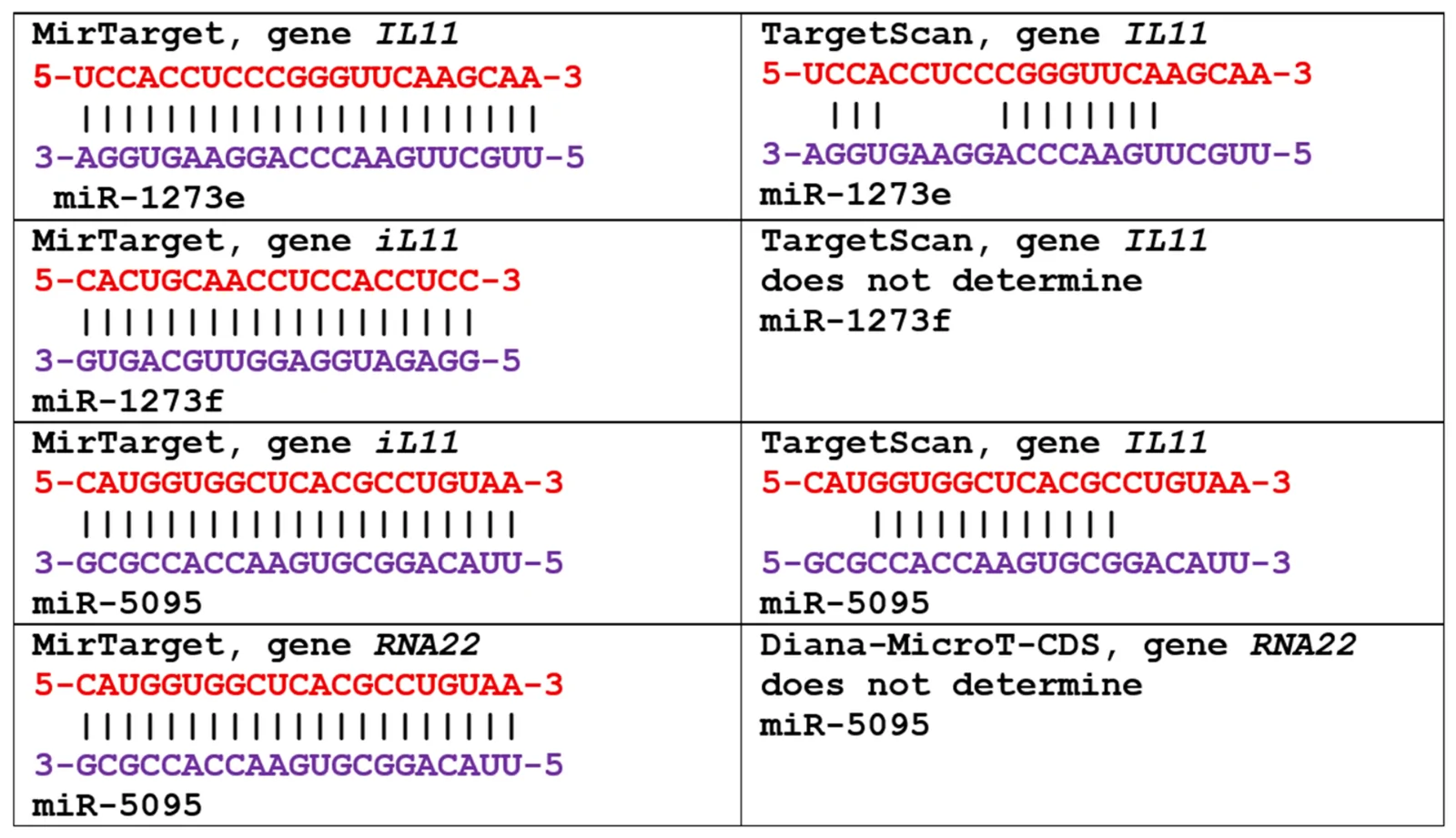
miRNA–mRNA interaction networks predicted using various software programs. Note: The mRNA nucleotides are highlighted in red. The miRNA nucleotides that form canonical pairs with mRNA are highlighted in violet.

**Figure 2:**
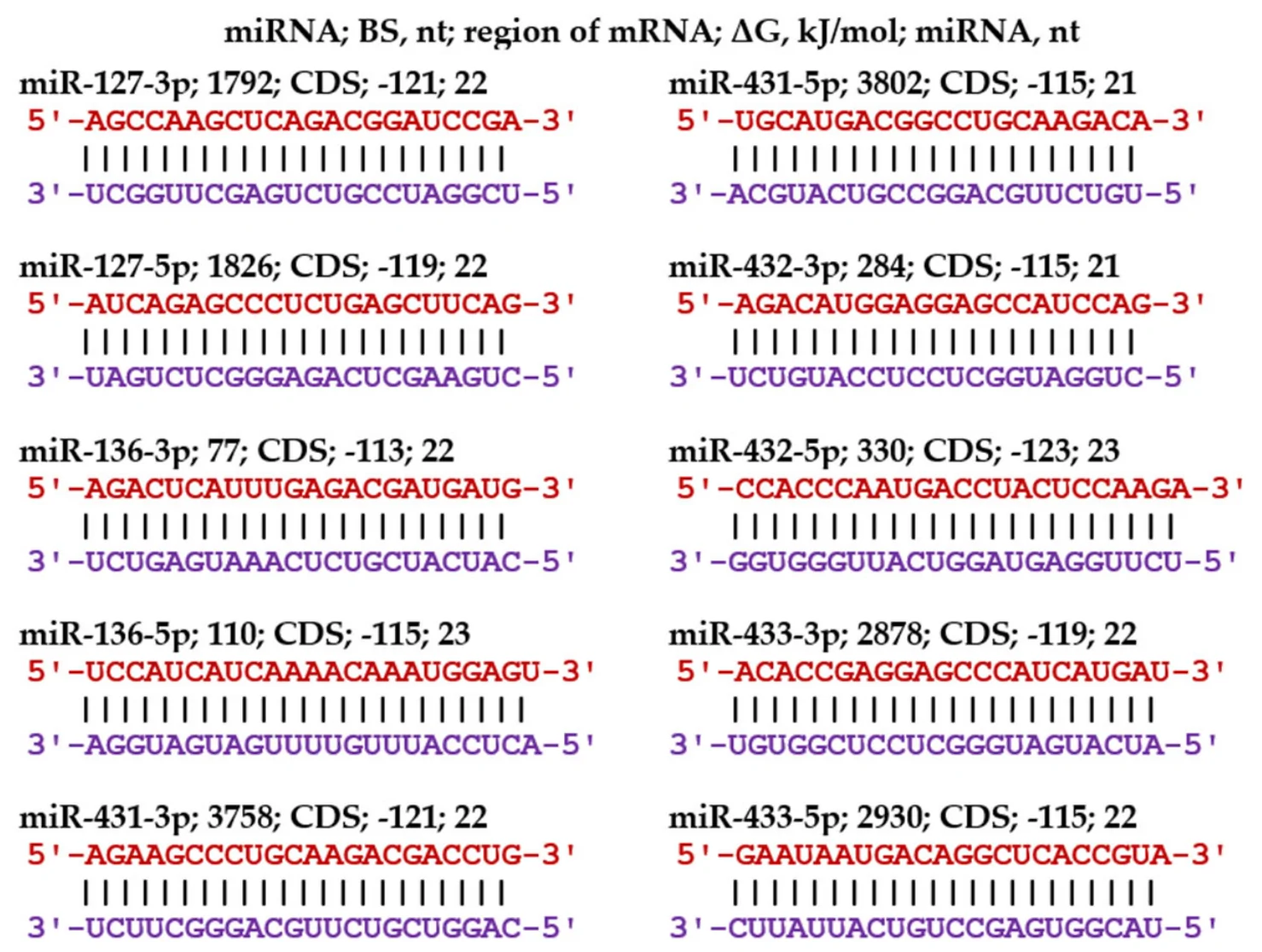
Schemes of interaction of miRNA nucleotides with mRNA of the *RTL1* gene. The mRNA nucleotides are highlighted in red. The miRNA nucleotides that form canonical pairs with mRNA are highlighted in violet.

**Figure 3:**
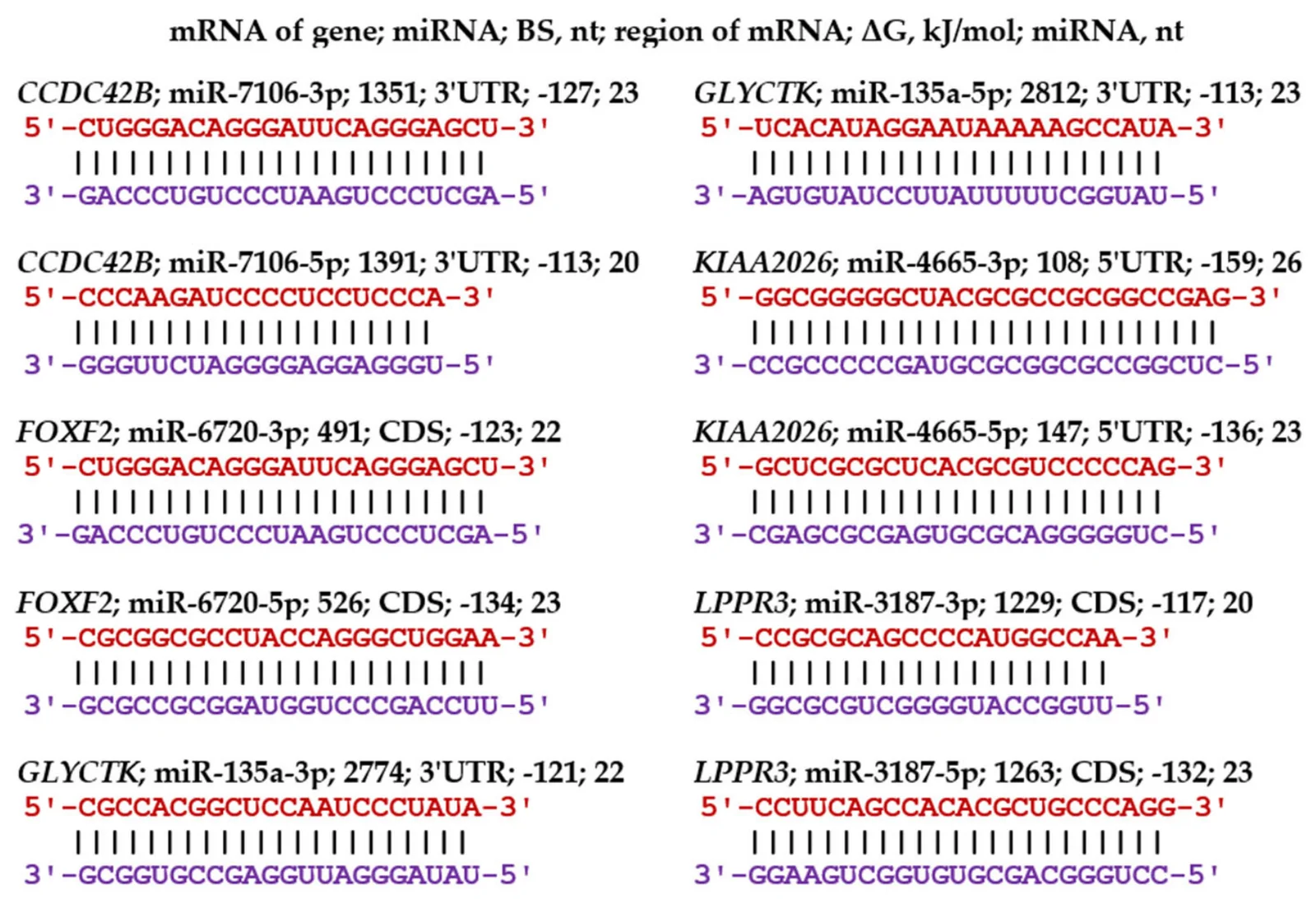
Schemes of interaction of miRNA nucleotides with mRNA of the *CCDC42B*, *FOXF2*, *GLYCTK*, *KAA2026* and *LPPR3* genes. The mRNA nucleotides are highlighted in red. The miRNA nucleotides that form canonical pairs with mRNA are highlighted in violet.

**Table 1:** miR-619-5p binding sites in target mRNAs.

mRNA of Gene	Binding Site, nt	mRNA of Gene	Binding Site, nt	mRNA of Gene	Binding Site, nt	mRNA of Gene	Binding Site, nt
*CSL6*	4638	*DAP3*	1841	*MR1*	3663	*SPATS2*	3331
*ADAL*	2040	***DCAF10***	3304	*MREG*	1539	*SPN*	5286
*ADAM17*	3465	***DCAF10***	4558	*MRPS25*	1608	*STAC2*	2240
*AGMAT*	2206	*DCLRE1C*	2965	*NDUFAF7*	1696	***SYNJ2BP***	1297
*AK1*	1448	*DDOST*	1781	*NDUFC2*	1645	***SYNJ2BP***	4174
*AKT2*	4570	*DHODH*	1708	*NLN*	4214	*TCEB1*	1963
*ALDH3A2*	2616	*DHRS9*	1280	*NRIP2*	2074	*TIGD6*	3438
*ANKRD16*	2164	*DNAJC22*	1553	*NSL1*	3062	*TMEM156*	1592
*AP5B1*	4315	*DNAL1*	4924	*NXPE3*	7446	*TMEM19*	3509
*ARGFX*	2641	*ERBB3*	5103	*OPTN*	2331	***TMEM213***	874
*ARHGEF39*	1306	*FADS6*	1776	*PAG1*	8155	***TMEM213***	1189
*ARL11*	1032	*FAM161A*	2784	*PAQR5*	4438	*TMEM50B*	1025
*ATCAY*	2990	*FAM227A*	4980	*PARK2*	3728	*TMEM56*	1242
*ATP1A2*	4409	*FAM84B*	3625	*PBLD*	2076	*TMF1*	4735
*BCL2L15*	2649	*FBLIM1*	2125	*PCGF5*	5088	*TMOD2*	7815
*BPNT1*	1127	*FBXL22*	1410	*PCSK5*	8612	*TNFRSF10A*	1620
*C15orf40*	522	*FBXO27*	1534	*PDAP1*	1925	*TNFRSF10D*	1531
***C17orf75***	2894	*FGD4*	7618	*PDCD4*	3220	*TOP3A*	3813
***C17orf75***	3671	***FKBP14***	1514	*PEX2*	3055	*TPRG1L*	1753
*C21orf58*	2667	***FKBP14***	2128	*PGPEP1*	1475	*TRIM72*	1884
*C4orf19*	2067	*FKBP5*	7113	*PIK3R2*	3344	***TRPM7***	8078
*C6orf170*	4112	*FXN*	3287	*PNPLA1*	1990	***TRPM7***	8220
***C8orf44***	335 *	*GDPD1*	1558	*PODNL1*	1875	*TXNDC15*	2459
***C8orf44***	1625	*GEMIN8*	2171	*POFUT1*	4678	*TYW5*	3691
*C9orf85*	870	*GGT6*	1955	*POLH*	5549	*UACA*	6119
*CACNB2*	4300	***GK5***	3807	*PPM1K*	2191	*UBIAD1*	2880
***CACNG8***	3217	***GK5***	6354	*PPP1R12B*	5155	*UBXN2A*	1664
***CACNG8***	5005	*GLB1L*	2223	*PRRG4*	997	*UPK1B*	1512
***CACNG8***	7534	*GOLGA3*	7239	*PSMB2*	2924	*UQCRB*	1268
*CALHM1*	2895	*GP2*	1876	*PTCD3*	4115	*USP29*	1 **
*CCBE1*	3320	*GPR65*	3308	*PTK6*	2232	***VHL***	3763
*CCDC114*	260 **	*GPR82*	2663	*QRFPR*	1948	***VHL***	3897
*CD109*	6840	*GPRIN2*	6675	*RAB11FIP1*	4927	*VWA2*	3365
*CD36*	4041	*GTPBP10*	1872	***RAB3IP***	3974	*WDR73*	1735
*CD68*	1397	*H6PD*	5753	***RAB3IP***	7021	***XIAP***	5680
*CDAN1*	4295	*HM13*	1744	*RAB7L1*	1692	***XIAP***	5814
*CDHR3*	4877	*IFIT3*	1863	*RBBP9*	1817	*YAE1D1*	1547
*CEP68*	4393	*ISY1*	685 *	*RGS3*	204 **	*ZBTB24*	4841
*CHST5*	2945	*IYD*	1657	*RPS6KA6*	7135	*ZC3H12D*	2811
***CHST6***	2978	*KIAA1456*	2535	*SCN11A*	5870	*ZDHHC20*	3389
***CHST6***	3875	*KIF11*	3597	*SEPT11*	4032	*ZFP30*	3462
***CIAO1***	2415	*KLHL23*	2569	*SEPT14*	1574	*ZNF114*	1826
***CIAO1***	3813	*KPNA1*	5710	*SGTB*	3141	*ZNF197*	3445
*CLEC19A*	1746	***KREMEN1***	2198	*SH3GLB1*	4855	*ZNF320*	5533
*CLTC*	7005	***KREMEN1***	2791	*SLC15A2*	4332	*ZNF429*	2080 *
*CORO2A*	2226	*LAX1*	2056	*SLC17A5*	2388	*ZNF445*	8819
*COX18*	1263	*LILRA6*	2200	*SLC26A2*	5065	*ZNF461*	3086
***CPM***	2697	*LIMD1*	5734	*SLC26A4*	4209	*ZNF549*	3735
***CPM***	4995	*LIMS1*	3930	*SLC28A2*	2195	*ZNF557*	4790
*CPT2*	2556	***LMOD3***	3223	*SLC7A11*	6303	*ZNF626*	4619
*CYB5RL*	3425	***LMOD3***	3992	*SLC7A14*	8486	*ZNF667*	3239
***CYP20A1***	2538	*METTL6*	1187	*SNX22*	901	*ZNF716*	2798
***CYP20A1***	4708	*MR1*	3663	*SOWAHC*	3416	*ZNF780B*	5414
*CYP27C1*	3822	*MREG*	1539	*SPATA13*	5019	*ZNF84*	4919
*CYP2W1*	2175	*MRPS25*	1608	*SPATA5*	5647	*ZNF841*	3421

Note. Genes containing two miR-619-5p binding sites are shown in bold. *—CDS, **—5′UTR.

**Table 2:** miR-5096 binding sites in mRNA target genes.

mRNA of Gene	Binding Site, nt	mRNA of Gene	Binding Site, nt	mRNA of Gene	Binding Site, nt	mRNA of Gene	Binding Site, nt
*AHI1*	4538	*FAM73A*	5068	***LMOD3***	4066	*SLC25A15*	2854
*BHMT2*	1833	*GLTP*	1486	***MREG***	1611	***SPN***	6092
***C15orf40***	596	*HEATR5A*	6835	*MYO3B*	5488	*SRSF8*	3281
*CABP4*	2001	*HES2*	3503	*NME6*	981	*TCTA*	990
***CCBE1***	3096	*IAPP*	875	*NUBPL*	1942	*TTF1*	2806
*COG7*	2715	*IL17RD*	8084	*PEX5L*	5030	*XPR1*	5193
*CORO2A*	2389	*IL18*	891	*PLCXD1*	1807	*ZNF43*	4342
***CPM***	1900	*IL18*	903	***PNPLA1***	2064	***ZNF197***	3519
***DCAF10***	3376	***KIAA1456***	5136	*POU6F1*	3494	*ZNF234*	4129
*DCP1A*	4695	*KLHL8*	4381	*PPP2R1B*	3053	*ZNF714*	6670
*DCP2*	9185	*LARS*	4597	*RBM3*	1152		
***DNAL1***	2389	***LIMD1***	5836	*RFWD3*	4441		

Note. Genes containing two miR-619-5p binding sites are shown in bold.

**Table 3:** Interaction characteristics of target mRNAs with miR-1-155-3p and miR-1-356-5p family members.

mRNA of Gene	miRNA	Binding Site, nt	ΔG, kJ/mol	Region of mRNA
*ASXL1-v-1*	miR-1-155-3p	340	−138	5′UTR
*CPT1A-v-2*	miR-1-155-3p	97	−138	5′UTR
*CXXC4-v-1*	miR-1-155-3p	742	−138	CDS
*DISP2-v-1*	miR-1-155-3p	20	−138	5′UTR
*DNAJC21-*	miR-1-155-3p	62	−138	5′UTR
*ESPN-v-1*	miR-1-155-3p	1966	−138	CDS
*FAM131C-*	miR-1-155-3p	1	−138	5′UTR
*1HTT-v-1*	miR-1-155-3p	263	−138	CDS
*>IRX4-v-1*	miR-1-155-3p	1243	−138	CDS
*USP25-v-1*	miR-1-155-3p	183	−138	5′UTR
*ACTN2*	miR-1-356-5p	99	−146	5′UTR
*BMP8B*	miR-1-356-5p	250	−146	5′UTR
*BMP8B*	miR-1-356-5p	251	−146	5′UTR

**Table 4:** Interaction characteristics of target mRNAs with miR-548 family members.

mRNA of Gene	miRNA	Binding Site, nt	ΔG, kJ/mol	Region of mRNA
*KATNAL1*	miR-548aa	3945	−123	3′UTR
*TCEANC2*	miR-548aa	2658	−123	3′UTR
*KATNAL1*	miR-548am-5p	3534	−110	3′UTR
*LG10*	miR-548ap-3p	2116	−89	3′UTR
*ANKRD5*	miR-548ap-3p	3406	−89	3′UTR
*KATNAL1*	miR-548ap-3p	3501	−89	3′UTR
*TCEANC2*	miR-548ap-3p	2664	−89	3′UTR
*ANKRD5*	miR-548au-5p	3444	−104	3′UTR
*KATNAL1*	miR-548au-5p	3535	−104	3′UTR
*KATNAL1*	miR-548c-5p	3534	−110	3′UTR
*SMU1*	miR-548h-3p	6756	−115	3′UTR
*ANKRD5*	miR-548t-3p	3400	−123	3′UTR
*KATNAL1*	miR-548t-3p	3495	−123	3′UTR
*TCEANC2*	miR-548t-3p	2658	−123	3′UTR

**Table 5:** Characteristics of the interaction of miR-1273 families with mRNAs of target genes.

mRNA of Gene	miRNA	Binding Site, nt	ΔG, kJ/mol	Region of mRNA
*ACACB*	miR-1273f	7996	−104	3′UTR
*ATF7IP2*	miR-1273f	2817	−104	3′UTR
*CA13*	miR-1273f	2779	−104	3′UTR
*COX18*	miR-1273f	1851	−104	3′UTR
*GTPBP3*	miR-1273f	2143	−104	3′UTR
*KRAS*	miR-1273f	3208	−104	3′UTR
*LOC100996605*	miR-1273f	238	−104	CDS
*MAP3K13*	miR-1273f	4636	−104	3′UTR
*RNF125*	miR-1273f	2810	−104	3′UTR
*SLA2*	miR-1273f	1910	−104	3′UTR
*SPTSSA*	miR-1273f	1564	−104	3′UTR
*XPNPEP3*	miR-1273f	5392	−104	3′UTR
*AP3S2*	miR-1273g-3p	4111	−117	3′UTR
*C15orf38*	miR-1273g-3p	4433	−117	3′UTR
*C20orf203*	miR-1273g-3p	1938	−117	3′UTR
*CRLF3*	miR-1273g-3p	2030	−117	3′UTR
*EIF1AD*	miR-1273g-3p	2165	−117	3′UTR
*EVI5*	miR-1273g-3p	4673	−117	3′UTR
*GJC1*	miR-1273g-3p	4830	−117	3′UTR
*TRAF6*	miR-1273g-3p	4719	−117	3′UTR
*ZNF621*	miR-1273g-3p	4815	−117	3′UTR
*CASP10*	miR-1273h-5p	2622	−117	3′UTR
*GINS3*	miR-1273h-5p	442	−117	CDS
*MXRA7*	miR-1273h-5p	1379	−117	3′UTR
*ZNF445*	miR-1273h-5p	5930	−117	3′UTR

**Table 6:** Characteristics of the interaction of several mRNA target genes with one miRNA.

mRNA of Gene	miRNA	Binding Site, nt	ΔG, kJ/mol	Region of mRNA
*FBA1*	miR-1184	1016	−127	CDS
*FBA2*	miR-1184	1001	−127	CDS
*FBA3*	miR-1184	1001	−127	CDS
*ERP29*	miR-1260a	377	−100	CDS
*NGB*	miR-1260a	1118	−100	5′UTR
*MAML2*	miR-1260a	1715	−104	CDS
*SC5DL*	miR-1260a	4212	−108	3′UTR
*MAML2*	miR-1260b	1715	−104	CDS
*CENPL*	miR-1285-5p	2316	−113	3′UTR
*FAM73A*	miR-1285-5p	2974	−113	3′UTR
*LRAT*	miR-1285-5p	3475	−113	3′UTR
*TSN*	miR-1285-5p	1964	−113	3′UTR
*GPR179*	miR-4478	2280	−96	CDS
*KHSRP*	miR-4478	66	−96	5′UTR
*PRR12*	miR-4478	4396	−96	CDS
*RNF111*	miR-4478	2436	−96	CDS
*KANK3*	miR-4508	510	−106	CDS
*OXR1*	miR-4508	103	−106	5′UTR
*PXDNL*	miR-4508	2686	−106	CDS
*ALG1*	miR-4526	864	−127	CDS
*ALG1L2*	miR-4526	400	−127	CDS
*ITGAL*	miR-5095	4708	−117	3′UTR
*KANK4*	miR-5095	4753	−117	3′UTR
*RBBP4*	miR-5095	4279	−117	3′UTR

## Data Availability

The original contributions presented in this study are included in the article. Further inquiries can be directed to the corresponding authors.
